# Trends in Respiratory Syncytial Virus and Bronchiolitis Hospitalization Rates in High-Risk Infants in a United States Nationally Representative Database, 1997–2012

**DOI:** 10.1371/journal.pone.0152208

**Published:** 2016-04-06

**Authors:** Abigail Doucette, Xiaohui Jiang, Jon Fryzek, Jenna Coalson, Kimmie McLaurin, Christopher S. Ambrose

**Affiliations:** 1 Epidstat, Ann Arbor, MI, United States of America; 2 AstraZeneca, Gaithersburg, MD, United States of America; University of Tennessee Health Science Center, UNITED STATES

## Abstract

**Background:**

Respiratory syncytial virus (RSV) causes significant pediatric morbidity and is the most common cause of bronchiolitis. Bronchiolitis hospitalizations declined among US infants from 2000‒2009; however, rates in infants at high risk for RSV have not been described. This study examined RSV and unspecified bronchiolitis (UB) hospitalization rates from 1997‒2012 among US high-risk infants.

**Methods:**

The Kids’ Inpatient Database (KID) infant annual RSV (*ICD-9* 079.6, 466.11, 480.1) and UB (*ICD-9* 466.19, 466.1) hospitalization rates were estimated using weighted counts. Denominators were based on birth hospitalizations with conditions associated with high-risk for RSV: chronic perinatal respiratory disease (chronic lung disease [CLD]); congenital airway anomalies (CAA); congenital heart disease (CHD); Down syndrome (DS); and other genetic, metabolic, musculoskeletal, and immunodeficiency conditions. Preterm infants could not be identified. Hospitalizations were characterized by mechanical ventilation, inpatient mortality, length of stay, and total cost (2015$). Poisson and linear regression were used to test statistical significance of trends.

**Results:**

RSV and UB hospitalization rates were substantially elevated for infants with higher-risk CHD, CLD, CAA and DS without CHD compared with all infants. RSV rates declined by 47.0% in CLD and 49.7% in higher-risk CHD infants; no other declines in high-risk groups were observed. UB rates increased in all high-risk groups except for a 22.5% decrease among higher-risk CHD. Among high-risk infants, mechanical ventilation increased through 2012 to 20.4% and 13.5% of RSV and UB hospitalizations; geometric mean cost increased to $31,742 and $25,962, respectively, and RSV mortality declined to 0.9%.

**Conclusions:**

Among high-risk infants between 1997 and 2012, RSV hospitalization rates declined among CLD and higher-risk CHD infants, coincident with widespread RSV immunoprophylaxis use in these populations. UB hospitalization rates increased in all high-risk groups except higher-risk CHD, suggesting improvement in the health status of higher-risk CHD infants, potentially due to enhanced surgical interventions. Mechanical ventilation use and RSV and UB hospitalization costs increased while RSV mortality declined.

## Introduction

Respiratory syncytial virus (RSV) is a significant cause of morbidity in young children [1]. It is estimated that over 2 million children younger than 5 years of age in the United States require medical attention because of RSV each year [2]. RSV infections are more prevalent during the fall, winter, and spring months than summer months in most of the United States [3]. The initial RSV infection in a child is typically the most severe, with obstruction of small airways from epithelial necrosis, edema, and mucus production, but children remain susceptible to subsequent RSV infection after their primary infections [4]. RSV is the most common cause of bronchiolitis and pneumonia in children younger than 1 year [5]. Infants born preterm, and children with chronic lung disease (CLD), congenital heart disease (CHD), or other high-risk medical conditions have a higher risk of developing more severe RSV infections [6]. Little information is available about national trends among these groups at high-risk for RSV, and overall trends among all infants and children may not be representative of those among the much smaller higher-risk subgroups.

Inpatient hospital discharge data is useful for illustrating trends in severe disease caused by RSV and related respiratory diseases. Previous research by Hasegawa *et al*. [7] demonstrated a decrease in bronchiolitis hospitalizations in the United States from 2000 to 2009 among children younger than 2 years old and infants. However, these analyses were not specific to RSV infections, and trends in hospitalization rates among children with comorbidities that may increase their likelihood for RSV infections were not directly evaluated. The data source for these analyses was the Kids’ Inpatient Database (KID), a nationwide database of pediatric inpatient, outpatient, and emergency room hospital admissions developed for the Healthcare Cost and Utilization Project (HCUP) to understand cost and quality of health services, medical practice patterns, and other healthcare issues [8]. The present analysis also utilized KID to specifically evaluate recent trends in RSV and bronchiolitis hospitalization rates among infants at high-risk for RSV in the United States.

## Methods

A historical cohort study of infant (<1 year of age) RSV hospitalization rates was conducted using KID as the primary data set. The National Inpatient Sample (NIS) was used to help evaluate generalizability of the KID data. The KID and NIS are publically available databases. Patient records for the KID and NIS were anonymized and de-identified prior to public release for analysis. Analysis of de-identified data from the KID and NIS are exempt from federal regulations for the protection of human research participants. All procedures involving human participants and confidentiality were reviewed and approved by the Research Ethics Review Board of the Agency for Healthcare Research and Quality (AHRQ).

KID is a sample of pediatric discharges from more than 4100 US hospitals that participate in the HCUP [8], and representative national estimates of disease burden can be made using the discharge-level weight values supplied by KID. The KID captures up to 50 diagnosis and procedure codes for each hospital admission. Diagnoses were coded using the *International Classification of Diseases*, *Ninth Revision*, *Clinical Modification* (*ICD-9-CM*; hereafter, simply *ICD-9*).

Whereas one code was available to classify patients with RSV at the introduction of the *ICD-9* in 1979 (*ICD-9* 480.1: Pneumonia due to RSV), two additional RSV-specific codes were introduced in 1997 (*ICD-9* 466.11 and 079.6). Therefore, our analyses started in 1997 and extended through 2012, the most recent update of KID available for analysis, with new KID data available every three years. Any hospitalization with at least one of the three *ICD-9* codes (*ICD-9* 480.1, 466.11, and 079.6) was categorized as a hospitalization due to RSV. Although laboratory confirmation of the etiologic agent is not available in cases coded as being associated with RSV, these codes represent the most specific options for the identification of cases with disease related to RSV using ICD diagnostic coding alone. A previous study in US hospital-based emergency departments validated RSV-specific ICD-9 codes against independent, blinded laboratory testing and confirmed this high specificity, demonstrating laboratory confirmation of RSV in 87% of infants who were assigned RSV-specific ICD codes by the institutions [9].

Unspecified bronchiolitis (UB) hospitalizations were therefore evaluated as a comparison of a related outcome that is less specific to RSV. Although UB-coded hospitalizations include events resulting from RSV infections that are not identified because of limited RSV testing [10], UB-coded events also commonly represent bronchiolitis caused by pathogens other than RSV [9], UB hospitalizations included any hospitalization with a diagnosis code of 466.19 (acute bronchiolitis due to other infectious organisms) or 466.1 (acute bronchiolitis) without any RSV-specific diagnostic code.

Certain comorbidities place infants at higher risk of RSV infection [6]. Comorbidities of interest included CLD, CHD, Down syndrome without CHD, congenital airway anomalies, preterm births, and other rare congenital and metabolic comorbidities (Table 1). An RSV or UB hospitalization record with another *ICD-9* code indicating any of these conditions was considered to be comorbid for that condition. Whereas codes for preterm births were found on birth hospitalization records, they were rarely identified when non-newborn infants were hospitalized for RSV or UB; therefore, hospitalization rates in preterm infants could not be examined. Because CHD can vary significantly in clinical severity by diagnosis and there are a large number of infants born with CHD, the CHD population was further stratified into higher- and lower-risk categories based on a review of the incidence of RSV by individual *ICD-9* codes for CHD. If *ICD-9* codes for higher-risk CHD and lower-risk CHD were both associated with a hospitalization, the hospitalization was recorded as occurring in an individual with higher-risk CHD.

**Table 1:** *ICD-9-CM* Codes for Conditions Associated With High-Risk for Respiratory Syncytial Virus Disease and Weighted Estimates of Overall Infant Hospitalization from KID, 1997–2012.

High-Risk Condition^a^	*ICD-9-CM* Codes	Total Non-Birth Hospitalizations (N)^b^	Total Birth Hospitalizations (N)^b^
Chronic respiratory disease arising in the perinatal period (CLD)	770.7X	69185	48510
Congenital anomalies of the respiratory system	748.XX	65910	25297
Cystic fibrosis with pulmonary manifestations†	277.0X	5537	805
Higher-risk congenital heart disease	425.4X; 428.0X; 745.0X- 745.4X; 745.6X – 745.8; 746.01–746.5X; 746.7X – 746.85; 746.87; 747.1X; 747.21–747.49	214061	152262
Lower-risk congenital heart disease	745.5X; 745.9X; 746.00; 746.6X; 746.86; 746.89–746.9X; 747.0X; 747.20; 747.83	127566	263086
Neuromuscular disease†	330.XX; 335.XX; 343.XX; 356.XX; 358.1; 359.0X–359.23; 359.9; 775.2	10958	1369
Down syndrome without congenital heart disease	758.0X	13320	12549
HIV†	079.51, 079.52, 079.53	9	10
Immunodeficiency†	279.XX	10370	908
Congenital and metabolic†	740.XX; 741.XX; 742.XX; 754.2; 756.1X; 756.6; 758.1–758.9; 759.3; 759.7X – 759.9X; 271.0; 272.7; 277.5; 277.81; 277.82; 277.86	110566	68780

CHD, congenital heart disease; CLD, congenital lung disease; *ICD-9-CM*, *International Classification of Diseases*, *Ninth Revision*, *Clinical Modification*; KID, Kids’ Inpatient Database

^a^ If ICD codes for higher-risk CHD and lower-risk CHD were both associated with a hospitalization, the hospitalization was recorded as higher-risk CHD. Down syndrome without CHD is mutually exclusive of higher-risk CHD and lower-risk CHD. Furthermore, the other high-risk group is mutually exclusive of higher-risk CHD, lower-risk CHD, CLD, Down syndrome without CHD, and congenital airway anomalies; preterm birth could not be examined.

^b^ For the calculation of RSV rates by specific conditions, the numerator of the rate was the number of non-birth hospitalizations with the condition of interest who had RSV and the denominator was the number of birth hospitalizations with the condition of interest.

†Cystic fibrosis with pulmonary manifestations, neuromuscular disease, HIV, immunodeficiency, and congenital and metabolic were combined and analyzed as “Other High-risk Conditions” due to the small size of each population.

RSV and/or UB codes were included regardless of diagnostic code positioning, as limiting the analyses to hospitalizations with a primary diagnosis of RSV or UB may introduce bias if hospitals or specific doctors do not order diagnostic codes consistently. This was a particular concern given the high-risk population of interest, whose records must contain additional codes for severe comorbidities, and decisions about diagnostic code ordering for such patients may be more variable than with non-high risk populations.

Overall hospitalization rates for all infants were calculated using the weighted estimate of hospitalizations due to RSV or UB from the KID relative to estimates of all infant births by year. Annual counts of infant births were obtained from the Centers for Disease Control and Prevention (CDC) Wide-ranging Online Data for Epidemiologic Research (WONDER) [11–13]. Rates of infant hospitalizations by comorbidity category were estimated by determining the number of hospitalizations coded with both the comorbidity and RSV or UB, relative to the estimated number of births with that condition by year. To capture community-acquired RSV or UB, any records representing a newborn birth hospitalization (ie, any *ICD-9* code indicating newborn birth = V30.XX−V39.XX) were excluded from this calculation of cases. To estimate the number of infants born with the specified preexisting condition each year, we limited the KID data set to newborns (*ICD-9* codes V30.XX−V39.XX) and calculated the weighted frequency of newborn birth hospitalizations that included a code for each specific condition in a given year. Infants who died during the birth hospitalization were excluded. This approach assumes that the number of newborns discharged with each condition is similar to the number of infants with each condition in the given year. All rates were estimated per 1000 infants per year and were assessed for trends over time using unadjusted Poisson regression.

Hospitalizations for RSV and UB were further described by inpatient mortality, use of mechanical ventilation, length of stay (LOS), and total costs. Inpatient mortality (coded as “Died” in the KID) and mechanical ventilation use (procedure codes 9390, 9392, 9601, 9602, 9603, 9604, 9605, 9670, 9671, or 9672) were evaluated as a proportion of total hospitalizations. Unadjusted logistic regression was used to examine temporal trends in mechanical ventilation and inpatient mortality. Because lengths of hospitalization stay and total hospitalization charges were not normally distributed, geometric means were calculated. Cost estimates were inflation-adjusted to 2015 dollars [14]. Unadjusted linear regression was used to test temporal trends in lengths of hospitalization stay and total hospitalization charges. All data management and analyses for this study were performed using SAS/STAT software, version 9.3 of the SAS System (SAS Institute Inc., Cary, NC, USA), with statistical procedures that incorporated weights to account for the structure of the sample survey data.

To evaluate generalizability of the KID results, analyses were repeated in 2 additional nationally-representative databases. The NIS is a 20% sample of discharges from all community hospitals that participate in HCUP. It is the largest publicly available all-payer inpatient healthcare database in the United States and contains more than 7 million hospital stays for patients of all ages annually [15]. Although the NIS and KID may potentially obtain data from the same hospitals, the sampling structure was not necessarily designed to do so. More information on the differences and similarities of these databases can be found at the HCUP website (https://www.hcup-us.ahrq.gov/tech_assist/faq.jsp). The National Hospital Discharge Survey (NHDS) is a CDC-funded survey that was conducted annually from 1965 to 2010. Before 2008, approximately 500 hospitals contributed data to this data set each year; this decreased to 239 hospitals between 2008 and 2010 [16]. All of our analyses were repeated in the NIS. Data from the NHDS were used for the overall analyses of RSV and UB trends but had limited utility for comorbidity analyses because the sample was much smaller and estimates were unstable.

## Results

Nearly 4 million non-birth infant hospitalizations were included in these analyses (Table 2). Hospitalizations due to RSV and UB represented 12.3% (n = 461,625) and 8.3% (n = 313,471) of these hospitalizations, respectively. The distribution of RSV hospitalizations by individual *ICD-9* code was 5.2% *ICD-9* 079.6, 88.4% *ICD-9* 466.11, and 11.2% *ICD-9* 480.1 (a hospitalization could have more than one RSV ICD code associated with it, so the combined percentage for the specific RSV ICD codes exceeds 100%). UB hospitalizations were 99.4% *ICD-9* 466.19 and 0.6% *ICD-9* 466.1, with no hospitalizations using code 466.1 after 1997, the first year analyzed. In addition, 0.07% of all hospitalizations had both an RSV and UB code associated with the hospitalization; these were considered RSV hospitalizations for the analyses. RSV was the primary diagnostic code in 88.1% of RSV-coded hospitalizations among non-high-risk infants and 76.0% of RSV-coded hospitalizations among infants with comorbidities that put them at high-risk for RSV. UB was the primary diagnostic code in 80.9% of UB-coded hospitalizations among non-high-risk infants and 70.5% of high-risk infants. Compared to the birth population, hospitalizations overall and those specific to RSV and UB were more frequently observed among infants who were male, Hispanic, and had insurance other than private insurance, such as Medicaid, regardless of comorbidity risk. As expected, RSV and UB admission counts were more frequent in the first and fourth quarters compared with other months (Table 2). Overall, the proportion of infant bronchiolitis hospitalizations that were coded as RSV was consistent during the study period, ranging between 57% and 63% across study years.

**Table 2:** Patient Characteristics of Hospitalized Non-Birth Infants in KID, 1997 to 2012.

Characteristic		High-Risk for RSV				Non−High-Risk			
		Birth Hospital-izations	All Non-Birth Hospital-izations	Non-Birth RSV Hospital-izations	Non-Birth UB Hospital-izations	Birth Hospital-izations	All Non-Birth Hospital-izations	Non-Birth RSV Hospital-izations	Non-Birth UB Hospital-izations
Total hospitalizations from all 6 study years, n		524,372	532,363	23,709	21,494	22,839,139	3,233,681	437,916	291,978
% of total hospitalizations				4.5	4.0			13.5	9.0
Sex, %									
	Male	51.8	56.2	57.0	60.2	51.0	57.4	57.3	61.4
	Female	48.1	43.8	43.0	39.8	48.8	42.5	42.6	38.5
Race or ethnic group, %									
	White	40.4	39.3	36.2	33.5	43.2	38.7	39.9	35.3
	Black	15.4	12.4	14.0	16.9	10.7	12.6	11.6	14.6
	Hispanic	18.5	18.3	22.5	24.3	16.8	19.9	20.6	23.8
	Asian or Pacific Islander	3.0	2.3	1.8	2.3	3.5	2.4	1.6	1.8
	Native American	0.5	0.7	0.8	0.7	0.5	0.6	0.7	0.7
	Other	4.5	5.9	4.7	4.7	4.4	4.7	4.2	4.3
Admission quarter, %									
	January−March	21.6	24.7	55.3	39.7	22.2	30.8	59.2	44.0
	April−June	22.4	22.6	8.0	17.6	22.8	19.7	6.5	15.5
	July−September	23.2	22.1	2.4	9.3	24.3	18.0	1.3	6.8
	October−December	22.4	22.8	25.3	24.9	23.0	23.0	24.9	24.9
Type of health insurance, %									
	Private	48.6	40.5	33.7	30.2	51.8	38.8	37.2	32.4
	Other	51.2	59.3	66.2	69.7	48.0	60.9	62.6	67.4

KID, Kids’ Inpatient Database; RSV, respiratory syncytial virus; UB, unspecified bronchiolitis.

Rates of all-cause hospitalization among all non-birth infants decreased by 25.4% during the study period, from 176.57 per 1000 infants in 1997 to 131.81 per 1000 infants in 2012 (p_trend_<0.01). Rates of hospitalization due to RSV among all non-birth infants decreased by 12.3% during the study period, from 20.30 per 1000 infants in 1997 to 17.80 per 1000 infants in 2012 (p_trend_<0.01). Hospitalizations due to UB decreased by 31.9%, from 15.45 per 1000 in 1997 to 10.52 per 1000 in 2012 (p_trend_<0.01). Similarly, a reduction was observed for hospitalizations due to causes other than RSV and UB, with a 26.5% reduction from 140.83 per 1000 in 1997 to 103.48 per 1000 in 2012 (p_trend_<0.01).

During their birth hospitalizations, infants in high-risk categories had worse severity indicators than low-risk infants. Infants born with high-risk comorbidities for RSV had longer geometric mean LOS (higher-risk CHD = 4.9 days; lower-risk CHD = 6.9 days; CLD = 73.4 days; congenital airway anomalies = 6.0 days; other high risk = 2.2 days) compared with non-high-risk infants (LOS = 2.0 days) for the entire study period (1997‒2012), except among those with Down syndrome without CHD (LOS = 1.9 days). In addition, among all non-birth hospitalizations, a higher proportion of infants born with high-risk comorbidities had mechanical ventilation use after birth (all high-risk groups combined = 33.4% versus 2.4% among non-high-risk infants born) and more frequent inpatient mortality (4.5% versus 0.2%).

**Fig 1:**
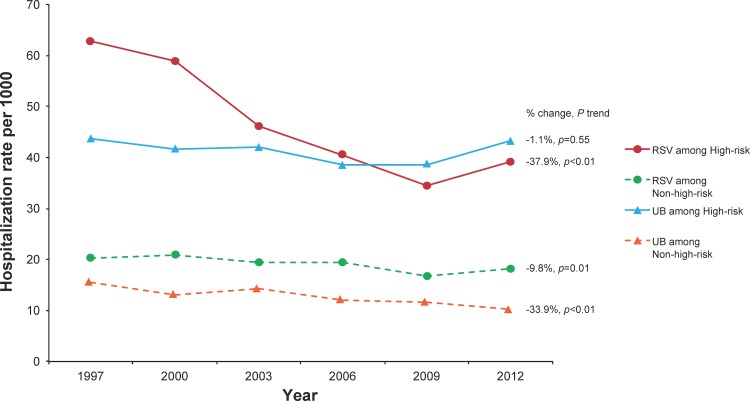
Rate of Hospitalizations due to Respiratory Syncytial Virus (RSV) or Unspecified Bronchiolitis (UB) According to High-Risk Status in KID, 1997–2012.

**Fig 2:**
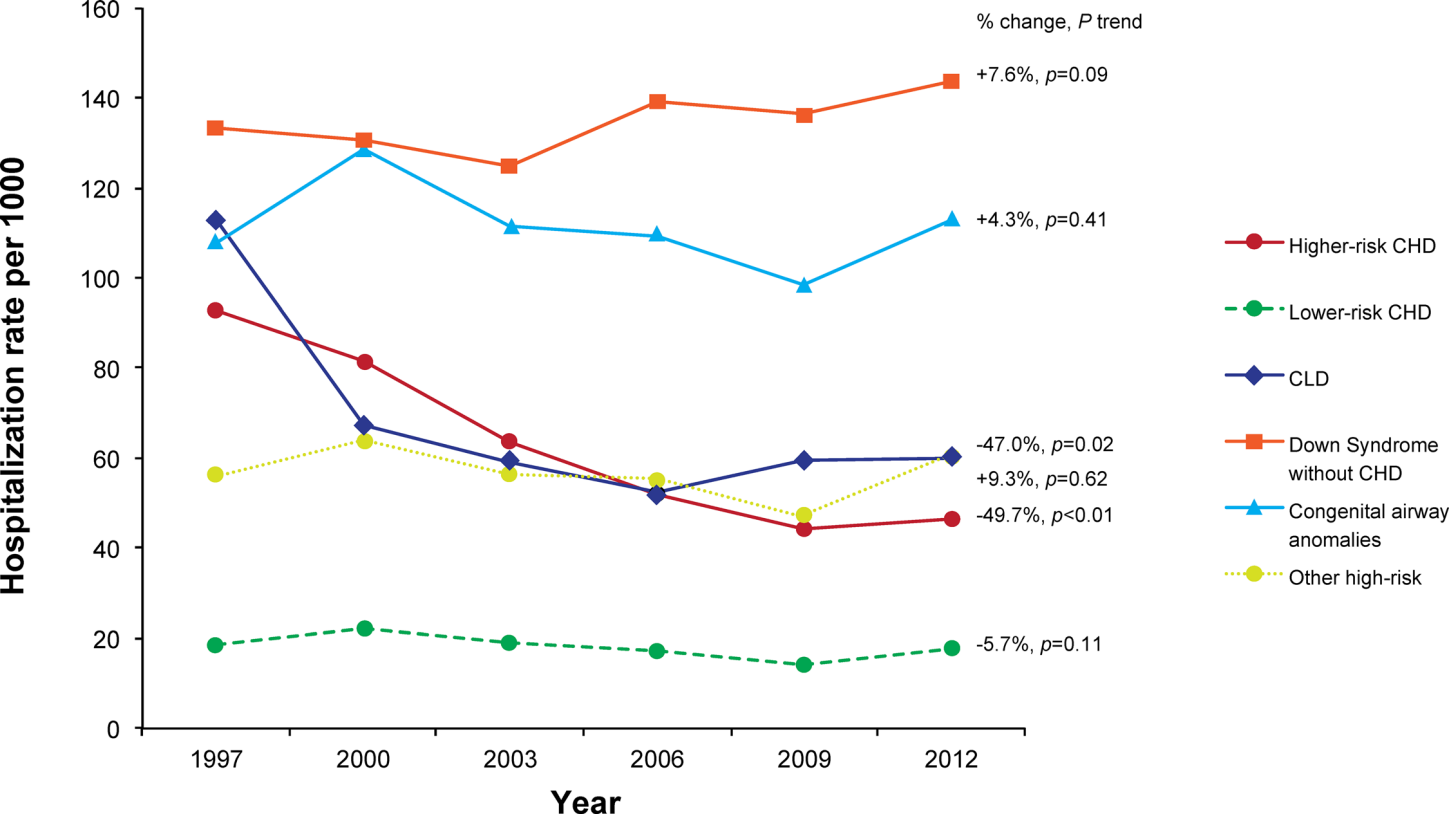
Hospitalization Rates due to Respiratory Syncytial Virus by High-Risk Comorbidities Among Infants in KID, 1997–2012.

**Fig 3:**
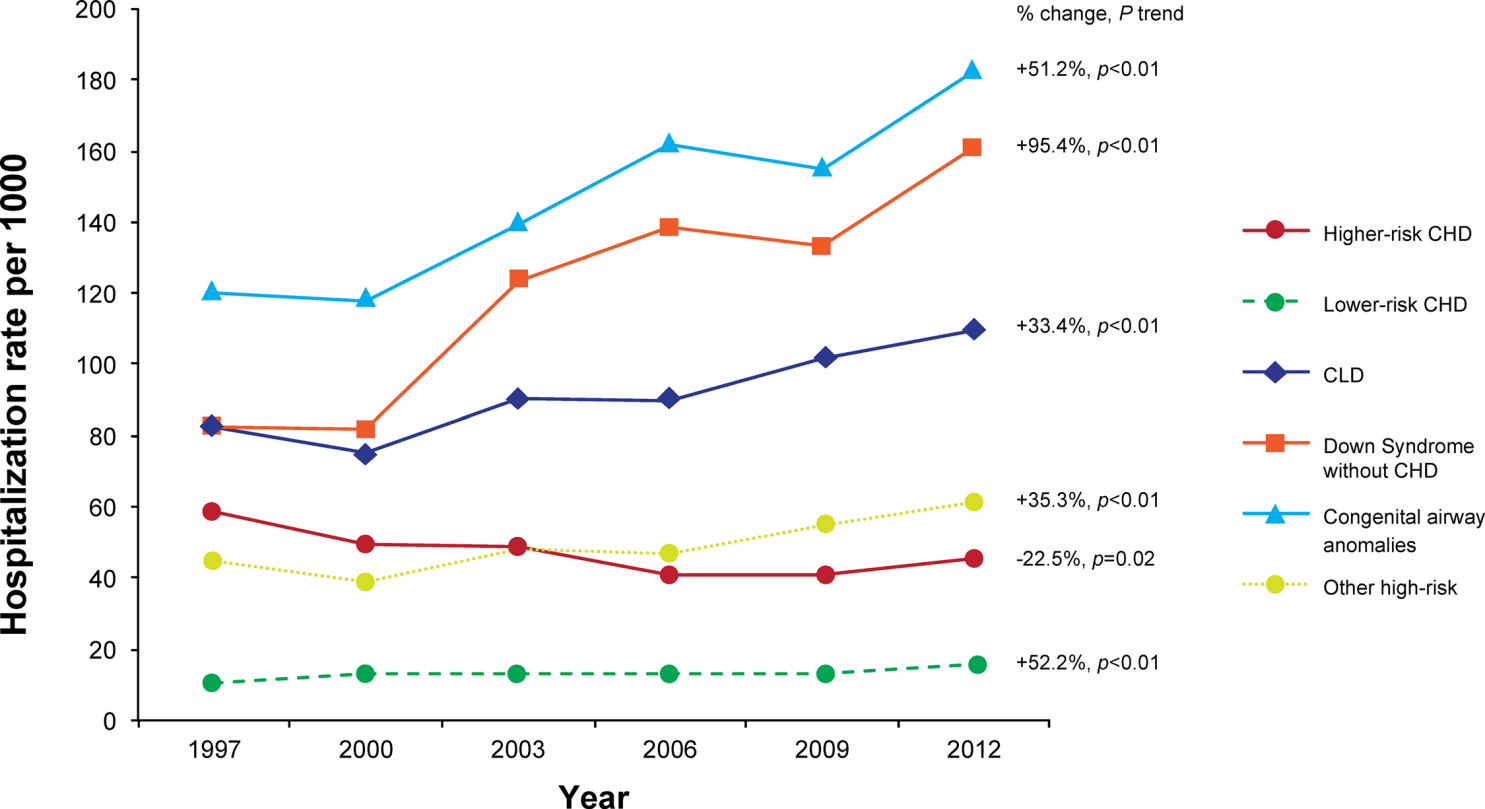
Hospitalization Rates due to Unspecified Bronchiolitis by High-Risk Comorbidities Among Infants in KID, 1997–2012.

Hospitalizations due to RSV among infants with any high-risk comorbid condition decreased by 37.9% during the study period, from a rate of 62.91 per 1000 in 1997 to 39.05 per 1000 in 2012 (p_trend_<0.01). The rates were primarily driven by ICD-9 code 466.11 (bronchiolitis due to RSV), as it comprised 88.4% of RSV-specific hospitalizations, but the trends were similar among hospitalizations that used the other RSV-specific codes (data not shown). Among this same group of infants, hospitalization rates due to UB remained stable across the study period, at 43.60 per 1000 in 1997 and 43.14 per 1000 in 2012 (p_trend_ = 0.55) (Fig 1). Among specific comorbidity groups, the hospitalization rate of RSV decreased by 47.0% among infants with CLD (p_trend_ = 0.02) and by 49.7% among those with higher-risk CHD (p_trend_<0.01), but no declines were seen in Down syndrome without CHD, congenital airway anomalies, lower-risk CHD, or other high-risk groups (Fig 2). The decline among CLD infants primarily occurred between 1997 and 2000, while the decline in higher-risk CHD primarily occurred between 1997 and 2006. In contrast to RSV rates, UB rates increased in all comorbidity groups except for higher-risk CHD, which decreased by 22.5% (p_trend_ = 0.02) over the study period (Fig 3). As would be expected based on the above results, when cumulative hospitalization rates for RSV or UB were evaluated, decreases were observed among CLD infants and higher-risk CHD infants, while increases were observed for all other high-risk groups.

To examine differences in hospitalized illness severity by high-risk status during the study period, inpatient mortality, mechanical ventilation use, LOS, and total charges were examined (Table 3 and Table 4). In general, RSV hospitalizations were more severe than UB hospitalizations. The percent of hospitalizations with inpatient mortality significantly decreased among high-risk and non-high-risk infants with RSV (p_trend_<0.01) (Table 3 and S1 Fig), but remained significantly elevated among those with high-risk comorbidities (0.9% in 2012) relative to those without (0.04% in 2012). In contrast, there was no significant decrease in mortality for patients with UB (Table 4 and S2 Fig). Similarly, the rate of inpatient mortality for high risk infants with RSV significantly decreased from 1.3 per 1000 infants in 1997 to 0.4 per 1000 infants in 2012 (p<0.01), but not for infants with UB, regardless of their high risk category (S1 Table and S2 Table).

**Table 3:** Hospitalized Illness Severity Indicators Among Infant Hospitalizations for Respiratory Syncytial Virus in the KID, 1997−2012.

		Mechanical Ventilation Use (% hospitalizations)			Inpatient Mortality*(% hospitalizations)			Length of Stay, d^a^^,^^d^			Total Charges, USD^a^^,^^b^		
		1997	2012	p_trend_	1997	2012	p_trend_	1997	2012	p_trend_	1997	2012	p_trend_
Groups at High-risk for RSV													
	Higher-risk CHD	17.4	18.9	0.07	2.1	1.5	0.18	5.78	5.32	0.72	19,236.28	31,917.23	<0.01
	Lower-risk CHD	19.9	25.6	<0.01	1.5	0.7	0.28	5.52	6.47	<0.01	20,475.26	40,131.32	<0.01
	CLD	27.1	32.2	0.12	2.6	0.6	0.18	8.24	8.68	0.79	28,473.96	55,286.56	<0.01
	Down syndrome without CHD	6.5	10.3	0.63	0	0	‒	3.67	4.00	0.32	10,141.30	18,217.61	<0.01
	Congenital airway anomalies	18.5	22.4	0.13	1.7	1.1	0.50	7.06	5.30	0.07	22,405.83	30,481.36	<0.01
	Other high-risk condition^c^	20.1	15.5	0.63	2.2	0.8	0.11	6.90	4.47	<0.01	21,679.81	26,206.74	<0.01
	Overall	18.7	20.4	0.01	2.0	0.9	<0.01	6.06	5.39	0.26	20,092.34	31,741.68	<0.01
Non−high-risk		3.0	4.5	<0.01	0.1	0.04	<0.01	2.82	2.42	<0.01	6,983.05	11,273.00	<0.01

CHD, congenital heart disease; CLD, chronic lung disease; KID, Kids’ Inpatient Database; RSV, respiratory syncytial virus

UB, unspecified bronchiolitis.

^a^ Geometric mean.

^b^ Adjusted to 2015 USD.

^c^ Other high risks: cystic fibrosis with pulmonary manifestations, neuromuscular disease, HIV, immunodeficiency, and other genetic metabolic musculoskeletal conditions.

^d^ A value of 0.01 was assigned to zero values in order to perform log-transformation.

* Small sample size.

If *ICD-9* codes for higher-risk CHD and lower-risk CHD were both associated with a hospitalization, the hospitalization was recorded as higher-risk CHD. Down syndrome without CHD is mutually exclusive of higher-risk CHD and lower-risk CHD. Furthermore, the other high-risk group is mutually exclusive of higher-risk CHD, lower-risk CHD, CLD, Down syndrome without CHD, and congenital airway anomalies.

**Table 4:** Hospitalized Illness Severity Indicators Among Infant Hospitalizations for Unspecified Bronchiolitis in KID, 1997−2012.

		Mechanical Ventilation Use(% hospitalizations)			Inpatient Mortality*(% hospitalizations)			Length of Stay, d^a^^,^^d^			Total Charges, USD^a^^,^^b^		
		1997	2012	p_trend_	1997	2012	p_trend_	1997	2012	p_trend_	1997	2012	p_trend_
Groups at High-risk for RSV													
	Higher-risk CHD	5.8	17.4	<0.01	0.5	1.4	0.17	3.31	4.88	<0.01	10,197.25	33,478.07	<0.01
	Lower-risk CHD	3.0	14.1	<0.01	0.8	0.3	0.65	3.18	4.48	<0.01	9008.58	27,248.36	<0.01
	CLD	7.7	15.5	<0.01	0.4	0.6	0.40	4.25	4.76	0.10	11,828.65	28,470.17	<0.01
	Down syndrome without CHD	0.8	5.3	<0.01	0	0.5	0.24	3.47	3.07	0.69	10,014.61	16,944.97	<0.01
	Congenital airway anomalies	4.8	14.5	<0.01	0	0.5	0.32	3.88	4.02	0.34	11,132.18	25,872.59	<0.01
	Other high-risk condition^c^	6.3	13.8	0.01	1.1	0.3	0.53	4.34	3.92	0.54	10,585.86	27,117.94	<0.01
	Overall	5.4	13.5	<0.01	0.4	0.7	0.39	3.67	4.11	<0.01	10,376.88	25,962.23	<0.01
Non−high-risk		0.7	2.4	<0.01	0.02	0.01	0.83	2.28	1.96	<0.01	5432.05	10,289.26	<0.01

CHD, congenital heart disease; CLD, chronic lung disease; KID, Kids’ Inpatient Database; RSV, respiratory syncytial virus

UB, unspecified bronchiolitis.

^a^ Geometric mean.

^b^ Adjusted to 2015 USD.

^c^ Other high risks: cystic fibrosis with pulmonary manifestations, neuromuscular disease, HIV, immunodeficiency, and congenital and metabolic conditions.

^d^ A value of 0.01 was assigned to zero values in order to perform log-transformation.

* Small sample size.

If *ICD-9* codes for higher-risk CHD and lower-risk CHD were both associated with a hospitalization, the hospitalization was recorded as higher-risk CHD. Down syndrome without CHD is mutually exclusive of higher-risk CHD and lower-risk CHD. Furthermore, the other high-risk group is mutually exclusive of higher-risk CHD, lower-risk CHD, CLD, Down syndrome without CHD, and congenital airway anomalies.

Mechanical ventilation use increased significantly from 1997 to 2012 in lower-risk CHD infants with RSV (p_trend_<0.01), primarily between 1997 and 2006, but the increase was not significant for higher-risk CHD infants (p_trend_ = 0.07) and the trends among other high risk groups were not clear (Table 3 and S1 Fig). Mechanical ventilation use increased significantly over time among non-high-risk infants with RSV and in all UB risk subgroups (all p_trend_<0.01) (Table 3; Table 4; S1 Fig; S2 Fig).

The geometric mean LOS was higher among high-risk infant hospitalizations due to RSV in 2012 compared with non-high-risk infant hospitalizations due to RSV (5.4 vs 2.4 days) (Table 3). A similar difference was seen for UB hospitalizations, with LOS of 4.1 and 2.0 days, respectively (Table 4). The geometric mean LOS for RSV hospitalizations significantly decreased for non-high risk infants over the study period, but high-risk subgroups hospitalized for RSV did not show a clear trend (S1 Fig). LOS of UB hospitalizations also decreased significantly among non-high risk infants over the study period, whereas the LOS among high-risk infants hospitalized due to UB significantly increased from 3.7 days in 1997 to 4.1 days in 2012, with the increase primarily occurring among infants with higher- and lower-risk CHD (Table 4; S2 Fig).

In 2015 dollars, the geometric mean cost for high-risk infant hospitalizations in 2012 was 2.8-fold and 2.5-fold higher than for non-high-risk infant hospitalizations for RSV and UB, at $31,742 and $25,962, respectively, for high-risk and $11,273 and $10,289, respectively, for non-high-risk. The cost of both RSV and UB hospitalizations in all risk groups and non-risk infants increased significantly between 1997 and 2012 (Table 3; Table 4; S1 Fig; S2 Fig).

The analyses presented above were repeated using the NIS and NHDS data sets where possible. Analyses in the NHDS were not stratified by risk category due to its small sample size. NHDS results generally supported the KID-based findings that trends in RSV hospitalizations decreased over time, but the results were less stable. NIS analyses were more stable for all infants and infant subgroups hospitalization rate estimates. Trends were similar in the NIS and KID among all comorbidity groups. The NIS confirmed the statistically significant decreases in RSV hospitalizations among infants with CLD and higher-risk CHD; however, estimated numbers and rates of RSV and UB hospitalizations among infants with CHD were slightly higher from the NIS.

## Discussion

The current analysis demonstrates that US all-cause hospitalization rates among non-birth infants have significantly decreased by 25.4% from 1997 to 2012, including declines in hospitalizations associated with RSV, UB, and other causes.

Contrary to the observed trends in all infants, RSV hospitalization rates among infants with comorbid conditions that put them at high-risk for RSV declined in only two categories, infants with CLD and infants with higher-risk CHD, with decreases of 47.0% to 49.7%. No declines in RSV hospitalization rates were observed in other high-risk groups and the cumulative rate of RSV or UB declined in these two populations. Additionally, there was no change in the proportion of infant bronchiolitis hospitalizations coded as RSV. These findings suggest that the RSV hospitalization declines in infants with CLD and infants with higher-risk CHD cannot be explained by changes in RSV testing and/or changes in the use of RSV-specific codes in US infants between 1997 and 2012. The observation that substantial declines in RSV hospitalization rates were limited to infants with CLD between 1997 and 2000 and higher-risk CHD between 1997 and 2006 may be explained, at least in part, by the widespread use of palivizumab. Palivizumab was approved in the United States in 1998 for RSV immunoprophylaxis in preterm infants and children with CLD of prematurity (previously referred to as bronchopulmonary dysplasia) and in 2003 for children with CHD. Randomized, placebo-controlled trials in these populations demonstrated 39% to 45% reductions in RSV hospitalizations among palivizumab recipients [17, 18], and observational studies have similarly demonstrated low RSV hospitalization rates among populations receiving palivizumab [19–26]. After approval, palivizumab use was directed to those at highest risk of severe RSV disease, particularly among infants born with CHD [27], consistent with the observed decrease in RSV hospitalization rates among children with higher-risk CHD but lack of significant change among those with lower-risk CHD. However, given the ecological design of the current analysis, other causal or contributing factors may be responsible for the observed declines, and additional research is needed to verify this hypothesis.

Hospitalization rates due to UB, which was evaluated for context as a comparable disease state, increased between 1997 and 2012 in all high-risk infants, with the exception of a 22% decrease among those infants with higher-risk CHD. The cause of these increases in UB hospitalization rates is unclear from the current results. The increase in LOS and lack of decline in inpatient mortality suggest that the increased hospitalization rates are not due to more lenient criteria for UB hospitalization. A greater clinical appreciation of non-RSV causes of infant bronchiolitis may have led to UB being more frequently assigned as a diagnosis among high-risk groups relative to other less-specific respiratory diagnoses during the study interval [28–30]. It is also possible that the risk of severe non-RSV bronchiolitis increased among high-risk infants. The absence of a decline in UB hospitalizations among infants with CLD suggests that RSV hospitalization decline in this population was not due to a lower risk of bronchiolitis disease from a general improvement in the health status of CLD infants. The decrease in UB hospitalization rates observed among infants with higher-risk CHD is difficult to interpret. The concomitant increase in mean LOS (+1.6 days) in this population suggests that severity of hospitalized UB in this population increased during the study interval. Additionally, it is possible that the overall health status of a subset of these infants may have improved during the study period due to advances in infant cardiac surgical repair [31, 32], leading to a decrease in the number of admissions for severe bronchiolitis. As a result, palivizumab and surgical advances may have contributed to the observed RSV and UB hospitalization rate declines in high-risk CHD infants, and further research is needed to fully understand these trends.

One previous study used nationally representative data to examine trends in RSV hospitalization for infants with CLD [33] and found a decrease in RSV hospitalization of 48% between 1998 and 2008. Our study, with a more robust database, additional comparison groups, and a longer period of follow-up, confirmed these findings. Similar results from our analyses of the NHDS and NIS further support the generalizability of the current results. There are few other studies describing national RSV hospitalization rates in US high-risk infants, but the overall rates of RSV hospitalizations among all non-birth infants from the KID observed in our study (20.30 per 1000 infants in 1997 to 17.80 per 1000 infants in 2012) are similar to those reported in other studies. Specifically, a recent meta-analysis by Nair (2010) [34] of population-based studies on RSV among infants published between 1995 and 2009 included 12 US studies. Excluding 4 studies conducted exclusively in Native American or Alaskan populations that have been shown to be at elevated risk of severe RSV disease, the annual incidence of inpatient RSV among US infants based on passive hospital case ascertainment ranged from 10 to 63 per 1000, with a median rate of 16.5 per 1000. A more recently published study based on the NHDS also reported that the rate of RSV-coded hospitalization among all infants ranged from 26.3 to 23.4 per 1000 between 1997 and 2006 [35]. All of these estimates, including those observed in the present study, will underestimate the true burden of RSV disease because of passive case ascertainment, limited testing and coding for RSV, and use of RSV tests with suboptimal sensitivity [34]. Stockman *et al*. estimated that the true annual infant hospitalization rates associated with RSV may be closer to 32 per 1000, by attributing 30% of winter bronchiolitis hospitalizations and 20% of winter pneumonia hospitalizations to RSV [35].

In addition to the observed changes in RSV and UB hospitalization rates, this analysis also provides information on the healthcare utilization and costs associated with RSV and UB hospitalizations in infants at high-risk for RSV and non-high-risk infants as well as the relative risk of RSV and UB in infant populations with various high-risk comorbidities. The results underscore the potential severity, mortality, and cost associated with RSV and UB hospitalizations, particularly among high-risk infants. The dramatic increases in the cost of RSV and UB hospitalizations among high-risk infants highlight the potential economic benefits of efforts to prevent these hospitalizations. Although children with CLD and higher-risk CHD have been the groups generally accepted to be at high-risk of RSV hospitalization, the current data also confirm the high-risk status of infants with congenital airway anomalies and Down syndrome without CHD, which has been demonstrated in smaller, previous studies [36–42].

One limitation of this analysis was the inability to include preterm infants, who are widely recognized as a high-risk group. Another primary limitation of the current analysis is the reliance on *ICD-9* coding for the outcomes of RSV and UB and the identification of comorbid conditions. Any substantial changes in national coding practices would affect the study results. However, the stability of many of the observed rates supports the conclusion that the observed trends are real and not primarily due to variability in clinical coding practices. Further, our analysis of UB as a comparison condition to RSV was designed to address the presence of any such trend as well as other temporal confounders. For our analyses of hospitalizations due to RSV and UB in children with certain comorbidities, we estimated the number of infants with the comorbidity of interest by calculating the number of newborns with the comorbidity. The accuracy of this method may vary by preexisting condition, as it assumes that all infants with the condition are diagnosed by the time of discharge from their birth hospitalization, and that postnatal mortality is low. Temporal changes in diagnosis or postnatal mortality could influence the observed trends in rates among high-risk infants. However, the comorbidities of interest are generally severe and highly likely to be diagnosed in the newborn hospital stay. Furthermore, if postnatal mortality has declined over time, we would expect the population at risk to have been disproportionately overestimated in the early years of the study, leading to disproportionate underestimation of the rates of RSV hospitalization in those groups at the beginning of the study period. In light of this, the observed decrease in rates of RSV hospitalizations among infants with CLD and higher-risk CHD are particularly striking.

## Supporting Information

S1 Fig Trends in Hospitalized Illness Severity Indicators among RSV Hospitalizations in KID Non-Birth Infants, 1997–2012. (A) Mechanical Ventilation Use (% Hospitalizations); (B) Inpatient Mortality (% Hospitalizations); (C) Length of Stay (days); (D) Total Hospital Charges (2015 US dollars).
(DOCX)

S2 Fig Trends in Hospitalized Illness Severity Indicators among UB Hospitalizations in KID Non-Birth Infants, 1997–2012. (A) Mechanical Ventilation Use (% Hospitalizations); (B) Inpatient Mortality (% Hospitalizations); (C) Length of Stay (days); (D) Total Hospital Charges (2015 US dollars).
(DOCX)

S1 Table Mortality Rate per 1000 Infants for Respiratory Syncytial Virus in the KID, 1997‒2012. (DOCX)

S2 Table Morality Rate per 1000 Infants for Unspecified Bronchiolitis in the KID, 1997‒2012. (DOCX)
