# Supplementary material for: Trends in Respiratory Syncytial Virus and Bronchiolitis Hospitalization Rates in High-Risk Infants in a United States Nationally Representative Database, 1997–2012
Source: PLoS One. 2016 Apr 6;11(4):e0152208. doi: 10.1371/journal.pone.0152208 (PMC4822775; doi:10.1371/journal.pone.0152208)
Supplement: S1 Fig — (A) Mechanical Ventilation Use (% Hospitalizations); (B) Inpatient Mortality (% Hospitalizations); (C) Length of Stay (days); (D) Total Hospital Charges (2015 US dollars). (DOCX) [file pone.0152208.s001.docx]

**S1 Figure. Trends in Hospitalized Illness Severity Indicators among RSV Hospitalizations in KID Non-Birth Infants, 1997–2012**

1. **Mechanical Ventilation Use (% Hospitalizations)**

|  | **Higher-risk CHD** | **Lower-risk CHD** | **CLD** | **Down Syndrome without CHD** | **Congenital airway anomalies** | **Other high risk** | **High risk** | **Non-high risk** |
| --- | --- | --- | --- | --- | --- | --- | --- | --- |
| 1997 | 17.44783 | 19.89757 | 27.05882 | 6.538702 | 18.46768 | 20.08013 | 18.73666 | 2.96829 |
| 2000 | 17.80916 | 12.26315 | 29.66368 | 12.38566 | 17.52342 | 13.9562 | 16.85569 | 3.389006 |
| 2003 | 18.06661 | 18.24747 | 31.0779 | 5.012254 | 21.63671 | 17.09649 | 17.89259 | 3.480842 |
| 2006 | 20.74952 | 25.77917 | 30.5409 | 8.055902 | 18.58715 | 14.70051 | 20.36913 | 3.707037 |
| 2009 | 21.93489 | 25.52624 | 33.54335 | 8.929368 | 23.80026 | 19.50825 | 21.76222 | 3.804537 |
| 2012 | 18.87369 | 25.59485 | 32.16873 | 10.33268 | 22.35616 | 15.4798 | 20.41342 | 4.545801 |
| p_trend_ | 0.07 | <0.01 | 0.12 | 0.63 | 0.13 | 0.63 | 0.01 | <0.01 |

1. **In-patient Mortality (% Hospitalizations)**

|  | **Higher-risk CHD** | **Lower-risk CHD** | **CLD** | **Down Syndrome without CHD** | **Congenital airway anomalies** | **Other high risk** | **High risk** | **Non-high risk** |
| --- | --- | --- | --- | --- | --- | --- | --- | --- |
| 1997 | 2.11457 | 1.47386 | 2.56544 | 0 | 1.65809 | 2.16530 | 2.00855 | 0.08360 |
| 2000 | 2.50433 | 1.12569 | 1.42134 | 0.48181 | 2.47933 | 1.65903 | 1.73724 | 0.07409 |
| 2003 | 2.04452 | 0.64334 | 1.86347 | 0 | 0.46277 | 3.71734 | 1.56063 | 0.06985 |
| 2006 | 1.62724 | 0.96866 | 0.75008 | 0 | 0 | 2.35346 | 1.17419 | 0.03688 |
| 2009 | 1.62834 | 0.69850 | 2.27775 | 0 | 1.83915 | 1.06649 | 1.19499 | 0.04636 |
| 2012 | 1.49431 | 0.71207 | 0.64133 | 0 | 1.14917 | 0.76030 | 0.93282 | 0.04043 |
| p_trend_ | 0.18 | 0.28 | 0.18 | - | 0.50 | 0.11 | <0.01 | <0.01 |

1. **Length of Stay (days)**

|  | **Higher-risk CHD** | **Lower-risk CHD** | **CLD** | **Down Syndrome without CHD** | **Congenital airway anomalies** | **Other high risk** | **High risk** | **Non-high risk** |
| --- | --- | --- | --- | --- | --- | --- | --- | --- |
| 1997 | 5.77968 | 5.520925 | 8.238294 | 3.666433 | 7.064059 | 6.897674 | 6.064607 | 2.819394 |
| 2000 | 5.602507 | 4.386498 | 7.960224 | 4.216457 | 5.48545 | 5.019123 | 5.322328 | 2.630438 |
| 2003 | 5.48083 | 5.32085 | 7.223152 | 3.832634 | 6.865176 | 5.753953 | 5.481045 | 2.522599 |
| 2006 | 5.582217 | 5.967077 | 6.888667 | 4.926288 | 5.474704 | 5.029032 | 5.495592 | 2.489552 |
| 2009 | 6.116234 | 5.986399 | 8.548161 | 4.286755 | 5.973257 | 5.871022 | 5.865925 | 2.466902 |
| 2012 | 5.320161 | 6.472248 | 8.680129 | 4.001233 | 5.299043 | 4.474213 | 5.392105 | 2.419444 |
| p_trend_ | 0.72 | <0.01 | 0.79 | 0.32 | 0.07 | <0.01 | 0.26 | <0.01 |

*Geometric mean

1. **Total Hospital Charges (2015 US dollars)**

|  | **Higher-risk CHD** | **Lower-risk CHD** | **CLD** | **Down Syndrome without CHD** | **Congenital airway anomalies** | **Other high risk** | **High risk** | **Non-high risk** |
| --- | --- | --- | --- | --- | --- | --- | --- | --- |
| 1997 | 19236.28 | 20475.26 | 28473.96 | 10141.3 | 22405.83 | 21679.81 | 20092.34 | 6983.052 |
| 2000 | 17521.19 | 14199.32 | 29387.3 | 11936.59 | 16632.09 | 14813.26 | 16743.3 | 6554.185 |
| 2003 | 22312.49 | 22755.8 | 31702.08 | 14584.31 | 26173.41 | 22342.02 | 22207.76 | 7791.043 |
| 2006 | 27622.2 | 29616.7 | 35307.94 | 18856.94 | 24215.55 | 22532.26 | 26026.07 | 8830.423 |
| 2009 | 32685.29 | 33482.22 | 50472.93 | 19174.65 | 30973.63 | 30561.5 | 31417.11 | 10045.2 |
| 2012 | 31917.23 | 40131.32 | 55286.56 | 18217.61 | 30481.36 | 26206.74 | 31741.68 | 11273 |
| p_trend_ | <0.01 | <0.01 | <0.01 | <0.01 | <0.01 | <0.01 | <0.01 | <0.01 |

*Geometric mean

**2015 US dollars
