# Supplementary material for: Trends in Respiratory Syncytial Virus and Bronchiolitis Hospitalization Rates in High-Risk Infants in a United States Nationally Representative Database, 1997–2012
Source: PLoS One. 2016 Apr 6;11(4):e0152208. doi: 10.1371/journal.pone.0152208 (PMC4822775; doi:10.1371/journal.pone.0152208)
Supplement: S2 Fig — (A) Mechanical Ventilation Use (% Hospitalizations); (B) Inpatient Mortality (% Hospitalizations); (C) Length of Stay (days); (D) Total Hospital Charges (2015 US dollars). (DOCX) [file pone.0152208.s002.docx]

**S2 Figure. Trends in Hospitalized Illness Severity Indicators among UB Hospitalizations in KID Non-Birth Infants, 1997–2012**

1. **Mechanical Ventilation Use (% Hospitalizations)**

|  | **Higher-risk CHD** | **Lower-risk CHD** | **CLD** | **Down Syndrome without CHD** | **Congenital airway anomalies** | **Other high risk** | **High risk** | **Non-high risk** |
| --- | --- | --- | --- | --- | --- | --- | --- | --- |
| 1997 | 5.84573 | 3.02924 | 7.729927 | 0.818031 | 4.822916 | 6.320862 | 5.405923 | 0.695515 |
| 2000 | 8.743391 | 3.906069 | 10.75854 | 0 | 9.112342 | 9.081193 | 7.586553 | 1.001716 |
| 2003 | 10.50951 | 6.152789 | 12.93484 | 0.535919 | 7.663155 | 5.077708 | 7.936264 | 1.104452 |
| 2006 | 13.00492 | 11.27902 | 12.63704 | 4.006174 | 11.70638 | 6.283037 | 10.26118 | 1.350184 |
| 2009 | 11.61011 | 10.73539 | 14.29912 | 6.917496 | 13.32961 | 9.94514 | 10.79007 | 1.611499 |
| 2012 | 17.44645 | 14.10781 | 15.48577 | 5.278195 | 14.46355 | 13.75529 | 13.5029 | 2.423372 |
| p_trend_ | <0.01 | <0.01 | <0.01 | <0.01 | <0.01 | 0.01 | <0.01 | <0.01 |

1. **In-patient Mortality (% Hospitalizations)**

|  | **Higher-risk CHD** | **Lower-risk CHD** | **CLD** | **Down Syndrome without CHD** | **Congenital airway anomalies** | **Other high risk** | **High risk** | **Non-high risk** |
| --- | --- | --- | --- | --- | --- | --- | --- | --- |
| 1997 | 0.474846 | 0.800039 | 0.3914 | 0 | 0 | 1.087095 | 0.412069 | 0.021906 |
| 2000 | 0.682402 | 0.324548 | 0.27303 | 0 | 0 | 1.37979 | 0.505682 | 0.017415 |
| 2003 | 1.231986 | 0 | 0 | 0 | 0.636363 | 1.36857 | 0.577875 | 0.021933 |
| 2006 | 0.683831 | 0.278636 | 0.55576 | 0.607052 | 0.376336 | 1.270278 | 0.453168 | 0.022052 |
| 2009 | 0.76043 | 0.197473 | 0.586956 | 0 | 0.196391 | 1.933615 | 0.543967 | 0.03276 |
| 2012 | 1.414799 | 0.316808 | 0.572253 | 0.519904 | 0.460649 | 0.280444 | 0.667174 | 0.014693 |
| p_trend_ | 0.17 | 0.65 | 0.40 | 0.24 | 0.32 | 0.53 | 0.39 | 0.83 |

1. **Length of Stay (days)**

|  | **Higher-risk CHD** | **Lower-risk CHD** | **CLD** | **Down Syndrome without CHD** | **Congenital airway anomalies** | **Other high risk** | **High risk** | **Non-high risk** |
| --- | --- | --- | --- | --- | --- | --- | --- | --- |
| 1997 | 3.314367 | 3.179705 | 4.254467 | 3.468374 | 3.879284 | 4.335305 | 3.667228 | 2.28335 |
| 2000 | 3.97657 | 3.264338 | 4.597168 | 3.28655 | 3.624266 | 4.23099 | 3.818331 | 2.137052 |
| 2003 | 3.715894 | 3.78197 | 4.509311 | 3.147019 | 3.77638 | 3.453418 | 3.655195 | 2.064431 |
| 2006 | 3.966257 | 3.736261 | 4.585471 | 3.582162 | 4.098344 | 3.425895 | 3.790137 | 2.039791 |
| 2009 | 4.359756 | 4.384959 | 4.925863 | 3.544462 | 4.100207 | 4.220477 | 4.176497 | 2.037421 |
| 2012 | 4.881461 | 4.47782 | 4.760554 | 3.071578 | 4.023088 | 3.916324 | 4.112881 | 1.964969 |
| p_trend_ | <0.01 | <0.01 | 0.10 | 0.69 | 0.34 | 0.54 | <0.01 | <0.01 |

*Geometric mean

1. **Total Hospital Charges (2015 US dollars)**

|  | **Higher-risk CHD** | **Lower-risk CHD** | **CLD** | **Down Syndrome without CHD** | **Congenital airway anomalies** | **Other high risk** | **High risk** | **Non-high risk** |
| --- | --- | --- | --- | --- | --- | --- | --- | --- |
| 1997 | 10197.25 | 9008.578 | 11828.65 | 10014.61 | 11132.18 | 10585.86 | 10376.88 | 5432.052 |
| 2000 | 12595.23 | 9511.792 | 13566.17 | 8630.521 | 11114.55 | 12108.3 | 11390.41 | 5418.901 |
| 2003 | 15765.96 | 14911.14 | 17367.85 | 12272.59 | 13572.07 | 13244.56 | 14394.07 | 6731.359 |
| 2006 | 19551.16 | 17933.1 | 19954.11 | 13736.77 | 18307.76 | 15118.38 | 17202.69 | 7754.965 |
| 2009 | 24482.42 | 24103.01 | 25787.28 | 16209.01 | 20129.88 | 21435.42 | 21836.48 | 9075.261 |
| 2012 | 33478.07 | 27248.36 | 28470.17 | 16944.97 | 25872.59 | 27117.94 | 25962.23 | 10289.26 |
| p_trend_ | <0.01 | <0.01 | <0.01 | <0.01 | <0.01 | <0.01 | <0.01 | <0.01 |

*Geometric mean

**2015 US dollars
