# Supplementary material for: Trends in Respiratory Syncytial Virus and Bronchiolitis Hospitalization Rates in High-Risk Infants in a United States Nationally Representative Database, 1997–2012
Source: PLoS One. 2016 Apr 6;11(4):e0152208. doi: 10.1371/journal.pone.0152208 (PMC4822775; doi:10.1371/journal.pone.0152208)
Supplement: S1 Table — (DOCX) [file pone.0152208.s003.docx]

**S1 Table.** Mortality rate per 1000 Infants for Respiratory Syncytial Virus in the KID, 1997‒2012

|  | | **Mortality rate (per 1000)** | | |
| --- | --- | --- | --- | --- |
|  |  | **1997** | **2012** | **p_trend_** |
| High-risk | Higher-risk CHD | 2.0 | 0.7 | <0.01 |
|  | Lower-risk CHD | 0.3 | 0.1 | <0.01 |
|  | CLD | 2.9 | 0.4 | 0.04 |
|  | Down syndrome without CHD | 0 | 0 | NA |
|  | Congenital airway anomalies | 1.8 | 1.3 | 0.51 |
|  | Other high-risk condition^a^ | 1.2 | 0.5 | 0.21 |
|  | Overall | 1.3 | 0.4 | <0.01 |
| Non−high-risk | | 0.02 | 0.01 | <0.01 |

CHD=congenital heart disease; CLD=chronic lung disease; HIV= human immunodeficiency virus; KID=The Kids’ Inpatient Database.

^a^Other high risks: cystic fibrosis with pulmonary manifestations, neuromuscular disease, HIV, immunodeficiency, and other genetic metabolic musculoskeletal conditions.
